# Divergent Effects of G2019S and R1441C LRRK2 Mutations on LRRK2 and Rab10 Phosphorylations in Mouse Tissues

**DOI:** 10.3390/cells9112344

**Published:** 2020-10-22

**Authors:** Lucia Iannotta, Alice Biosa, Jillian H. Kluss, Giulia Tombesi, Alice Kaganovich, Susanna Cogo, Nicoletta Plotegher, Laura Civiero, Evy Lobbestael, Veerle Baekelandt, Mark R. Cookson, Elisa Greggio

**Affiliations:** 1 Department of Biology, University of Padova, 35131 Padova, Italy; lucia.iannotta@unipd.it (L.I.); alice.biosa@autifony.com (A.B.); giulia.tombesi@phd.unipd.it (G.T.); kaganovichal@grc.nia.nih.gov (A.K.); susanna.cogo@unipd.it (S.C.); nicoletta.plotegher@unipd.it (N.P.); laura.civiero@unipd.it (L.C.); 2 Cell Biology and Gene Expression Section, National Institute on Aging, National Institutes of Health, Bethesda, MD 20892, USA; jillian.kluss@nih.gov (J.H.K.); cookson@mail.nih.gov (M.R.C.); 3 IRCCS San Camillo Hospital, 30126 Venice, Italy; 4 Laboratory for Neurobiology and Gene Therapy, KU Leuven, 3000 Leuven, Belgium; evy.lobbestael@kuleuven.be (E.L.); veerle.baekelandt@kuleuven.be (V.B.)

**Keywords:** LRRK2, phosphorylation, Rab10, mutant mice, striatum, cortex, midbrain, lung, kidney, age-dependent changes

## Abstract

Mutations in LRRK2 cause familial Parkinson’s disease and common variants increase disease risk. LRRK2 kinase activity and cellular localization are tightly regulated by phosphorylation of key residues, primarily Ser1292 and Ser935, which impacts downstream phosphorylation of its substrates, among which Rab10. A comprehensive characterization of LRRK2 activity and phosphorylation in brain as a function of age and mutations is missing. Here, we monitored Ser935 and Ser1292 phosphorylation in midbrain, striatum, and cortex of 1, 6, and 12 months-old mice carrying G2019S and R1441C mutations or murine bacterial artificial chromosome (BAC)-Lrrk2-G2019S. We observed that G2019S and, at a greater extent, R1441C brains display decreased phospho-Ser935, while Ser1292 autophosphorylation increased in G2019S but not in R1441C brain, lung, and kidney compared to wild-type. Further, Rab10 phosphorylation, is elevated in R1441C carrying mice, indicating that the effect of LRRK2 mutations on substrate phosphorylation is not generalizable. In BAC-Lrrk2-G2019S striatum and midbrain, Rab10 phosphorylation, but not Ser1292 autophosphorylation, decreases at 12-months, pointing to autophosphorylation and substrate phosphorylation as uncoupled events. Taken together, our study provides novel evidence that LRRK2 phosphorylation in mouse brain is differentially impacted by mutations, brain area, and age, with important implications as diagnostic markers of disease progression and stratification.

## 1. Introduction

Mutations in the gene encoding leucine-rich repeat kinase 2 (LRRK2) cause autosomal dominant Parkinson’s disease (PD), while common variants in the LRRK2 locus increase the lifetime risk of disease [1]. LRRK2 is a large signaling protein comprising serine-threonine kinase and ROC-COR GTPase catalytic domains as well as scaffolding modules [2]. PD-causing mutations are gain of function through a direct activating effect on kinase activity (G2019S) or by decreasing GTP hydrolysis (R1441C/G/H and Y1699C), which affects LRRK2 subcellular access to its substrates, the most validated being a subset of Rab GTPases [3]. LRRK2 controls its own phosphorylation through a yet unclear mechanism, likely involving heterologous kinases (CK1α, IKKs, PKA) and phosphatases (PP1) within a cluster of N-terminus residues, the two major ones being Ser910 and Ser935 (reviewed in [4]). Phosphorylation of these residues provides the docking sites for the binding of 14-3-3 proteins, which regulate LRRK2 activity and subcellular localization [5,6]. LRRK2 kinase inhibition results in LRRK2 dephosphorylation at Ser910/Ser935 and consequent dissociation from 14-3-3 proteins [4,5,6]. These residues are dephosphorylated also in several LRRK2 PD mutants with hyperactive kinase activity, raising a question as to whether dephosphorylated LRRK2 is pathogenic, protective or both, depending on the upstream mechanism that governs the dephosphorylation event.

Autophosphorylation of Ser1292 is a robust readout of LRRK2 kinase activity in cells [7], which was shown to positively correlate with Thr73 phosphorylation of Rab10, a well-established LRRK2 substrate [3]. As predicted, Ser1292-LRRK2 and Thr73-Rab10 are hyperphosphorylated in the presence of gain of function LRRK2 mutants in cells and mouse and human peripheral tissues [3,7,8,9], as well as in the substantia nigra (SN) dopaminergic neurons of postmortem brain tissue from idiopathic PD patients [10]. Despite being well-established in cells, Rab10 phosphorylation in the brain has proven difficult to detect, possibly due to the high levels of PPM1H, a phosphatase selectively dephosphorylating Rab GTPases [11], or to a lower basal activity of LRRK2 in neuronal cells compared to peripheral tissues.

To gain insights into the effects of LRRK2 mutations on protein activity and phosphorylation ex-vivo, we performed a comprehensive survey of LRRK2 and Rab10 phosphorylation in midbrain, striatum, and cortex, as well as in peripheral tissues of three LRRK2 mutant mice, namely knockin (KI) Lrrk2-G2019S, KI Lrrk2-R1441C, and BAC overexpressing murine Lrrk2-G2019S. We observed that KI G2019S animals exhibit elevated pSer1292 in brain and lung/kidney but no increased pThr73-Rab10 in lung/kidney as compared to wild-type (WT), whilst KI mice carrying the ROC R1441C mutation display pSer1292 levels surprisingly similar to WT brain/lung/kidney but increased pT73-Rab10. Phospho-Ser935 is barely detectable in KI R1441C brains, and halved in KI G2019S compared to WT. In mice overexpressing the murine Lrrk2 locus (BAC-Lrrk2-G2019S), total and phospho-LRRK2 levels increase in the aging striatum, while phospho-Thr73 Rab10 is decreased at 12 months, indicating that LRRK2 and Rab10 phosphorylations are uncoupled, at least in this model. Finally, histological evaluation of BAC-G2019S brains confirmed that Lrrk2 expression is considerably higher in the striatum compared to the cortex and further revealed that aged mice display elevated GFAP levels, suggestive of enhanced gliosis.

Taken together, our comprehensive ex-vivo study provides evidence that LRRK2 phosphorylation in murine brain is differentially impacted by mutations and that pSer1292/pSer935-LRRK2 and pThr73-Rab10 may be diagnostic of G2019S and R1441C-linked diseases, respectively.

## 2. Materials and Methods

### 2.1. Animals

C57BL/6 LRRK2 wild-type and mouse LRRK2 G2019S BAC (GS BAC) mice were obtained from Jackson Laboratory [B6.Cg-Tg(Lrrk2∗G2019S)2Yue/J]. Non-transgenic wild-type (WT) mice were littermates obtained from the heterozygous breeding. Housing and handling of mice were done in compliance with national guidelines. All animal procedures were approved by the Ethical Committee of the University of Padova and the Italian Ministry of Health (#200/2019-PR and 1041/2016-PR).

Homozygous LRRK2 G2019S knock-in (GSKI), R1441C knock-in (RCKI), knockout (KO) and parental WT mice were housed at the National Institute on Aging, NIH, [12,13] according to a protocol approved by the Institutional Animal Care and Use Committee of the National Institute on Aging, NIH (463-LNG-2019).

Dissections of cortex, midbrain, and striatal regions were performed in 1, 6, and 12 months-old mice of all genotypes. 4–5 mice/genotype/age were used in all experiments.

### 2.2. Antibodies

For western blotting the following antibodies were used: total LRRK2 [MJFF2 (c41-2)] (Abcam, Cambridge, UK, Cat# ab133474, 1:300), phospho-S935 LRRK2 (Abcam, Cambridge, UK, Cat# ab133450, 1:500), phospho-S1292 LRRK2 (Abcam, Cambridge, UK, Cat# ab203181, 1:300), total Rab10 (Abcam, Cambridge, UK, Cat# ab104859, 1:500), phospho-T73 Rab10 (Abcam, Cambridge, UK, Cat# ab230261, 1:400), tyrosine hydroxylase (Millipore, Burlington, MA, USA Cat# AB152, 1:10000), DARPP32 (Millipore, Burlington, MA, USA Cat# AB10518, 1:10000) and β-actin (Sigma-Aldrich, St. Louis, MO, USA Cat# A2066, 1:10000). For immunofluorescence: β-tubulin-III (Sigma-Aldrich, St. Louis, MO, USA, Cat# T8578, 1:1000); total LRRK2 [MJFF2 (c41-2)] (Abcam, Cambridge, UK, Cat# ab133474, 1:200), phospho-S935 LRRK2 [UDD2 10(12)] (Abcam, Cambridge, UK, Cat# ab172382, 1:200), GLT1 (EMD Millipore, Burlington, MA, USA Cat# AB1783, 1:400), GFAP (Dako-Agilent, Santa Clara, CA, USA, Cat# Z0334, 1:400) CD11b [M1/70] (eBioscience™ from Thermo Fisher Scientific, Waltham, MA, USA, Cat# 14-0112-82, 1:200).

### 2.3. Brain Lysis and Western Blotting

Brain regions were mechanically lysed in 25 mM pH = 7.5 Tris-HCl, 150 mM NaCl, 1% (*v/v*) NP40, 1% (*w/v*) sodium deoxycholate, 0.1% (*w/v*) SDS, 2 mM EGTA, 20 mM sodium fluoride, 50 mM beta glycerophosphate, 50 mM sodium pyrophosphate, 20 mM sodium orthovanadate. 70 μg of protein samples were resolved on pre-casted 4–20% Tris-glycine polyacrylamide gels (Biorad, Hercules, CA, USA).

Resolved proteins were transferred to polyvinylidenedifluoride (PVDF) membranes using semidry Biorad transfer machine (Trans-Blot Turbo Transfer System, Biorad, Hercules, CA, USA). After 1h of saturation in 0.1% Tween-20 (TBS-T) plus 5% non-fat dry milk, PVDF membranes were incubated overnight at 4 °C with specific primary antibodies. The PVDF sheets were washed in TBS-T (3 × 10 min) at room temperature (RT) followed by incubation for 1 h at RT with horseradish peroxidase-conjugated IgG. Immunoreactive proteins were visualized using chemiluminescence (Immobilon ECL western HRP substrate, Millipore, Burlington, MA, USA). Densitometric analysis was carried out using Image J software (U. S. National Institutes of Health, Bethesda, MD, USA). Levels of phospho-LRRK2/total LRRK2, phospho-RAB10/total RAB10 ratios were compared between control and mutant mice at different ages.

### 2.4. Immunofluorescence

Animals were terminally anesthetized with xylazine (Rompun^®^) and ketamine (Zoletil^®^) and transcardially perfused with 0.9% saline followed by ice cold 4% paraformaldehyde (PFA). Brains were dissected and post-fixed in 4% PFA at 4 °C overnight, then transferred to a sucrose gradient in phosphate-buffered saline (PBS) (20% and 30%) at 4 °C for cryopreservation. Once saturated in sucrose, 40 μm thick coronal slices were obtained by sectioning the brains with a vibratome. Sections were rinsed three times with PBS and then the sample autofluorescence was quenched in 50 nM NH_4_Cl in PBS. After three more washings, tissue sections were immersed in a solution made of 0.1% Sudan Black B (SBB) and 70% ethanol for 15 min at RT. To remove the excess of SBB the slices were rinsed three times with PBS and then they were permeabilized and saturated for 2 h in blocking solution (15% vol/vol goat serum, 2% wt/vol BSA, 0.25% wt/vol gelatin, 0.2% wt/vol glycine in PBS) containing 0.5% Triton X-100. Incubation with the primary antibodies was carried out overnight at 4 °C in blocking solution. Samples were washed three times with PBS and then sections were incubated with appropriate secondary antibodies diluted 1:200 in blocking solution. Images were acquired with Zeiss LSM700 confocal microscope, using 20×/0.8 M27 objective.

### 2.5. Statistical Analysis

All quantitative data are expressed as mean ± SEM (standard error of the mean) from at least 4 different mice/genotype/age. Significance of differences between two groups was verified by Student t-test while comparisons between 3 or more groups were performed by one-way analysis of variance (ANOVA) with Dunnett’s Multiple comparison test/Bonferroni’s post-hoc test.

## 3. Results

### 3.1. LRRK2 Expression in Midbrain, Striatum, and Cortex

**Figure 1:**
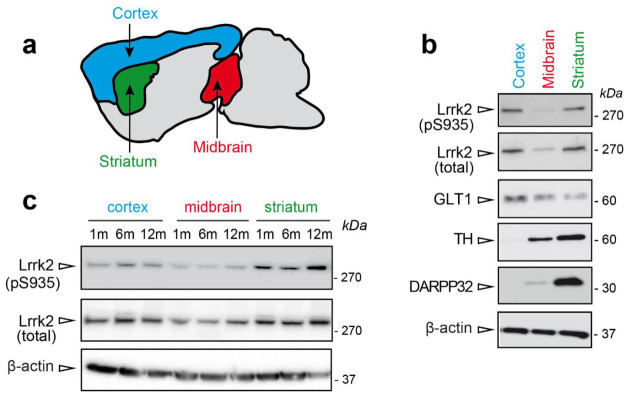
Lrrk2 steady state levels in Parkinson’s disease (PD)-relevant areas. (**a**) Schematic representation of cortex (blue), midbrain (red), and striatum (green) localization in mouse brain. (**b**) Representative western blot showing Lrrk2 steady state and phosphorylation levels in PD-relevant areas striatum, cortex, and midbrain of 24 months-old wild-type mice. (**c**) Lrrk2 steady state and phosphorylation levels in striatum, midbrain, and cortex of wild-type mice at different ages (1, 6 and 12 months-old).

To explore LRRK2 phosphorylation in mouse brain, we dissected three brain regions, namely striatum, cortex, and midbrain (Figure 1a). These areas are relevant in PD pathology [14] and LRRK2 was previously shown to be highly expressed in striatum and cortex [15,16]. Here we confirmed that Lrrk2 steady state levels are higher in the cortex and striatum compared to midbrain (Figure 1b), while the levels of phosphorylated Ser935 are higher in striatum compared to the other regions (Figure 1c). In addition, the lower Lrrk2 expression in the midbrain appears age-independent (Figure 1c).

### 3.2. Changes in Ser935 and Ser1292 Phosphorylation in G2019S and R1441C Knockin Brains at Different Ages

**Figure 2:**
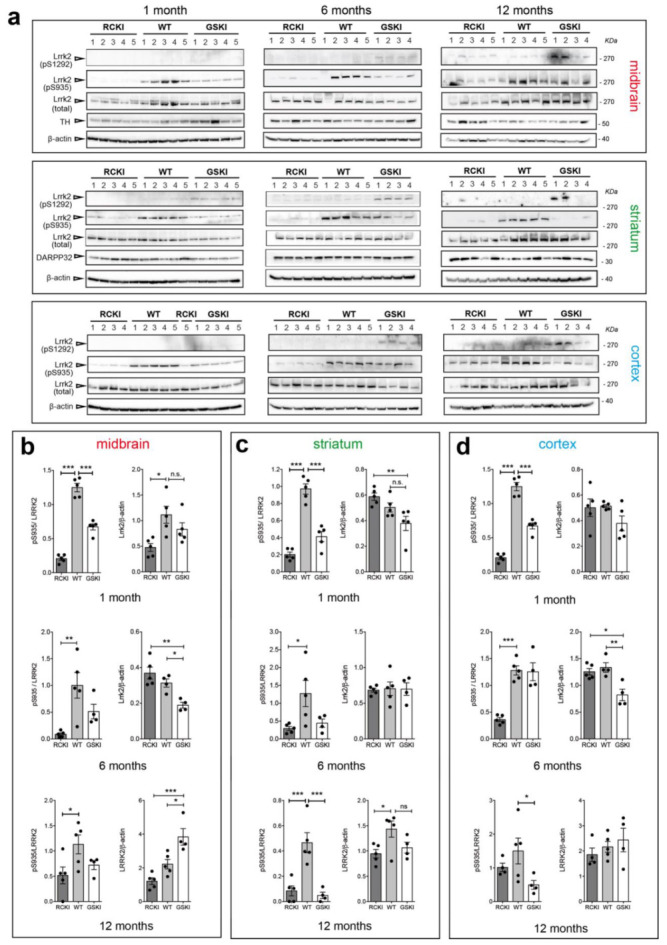
Age-related changes in Ser935 and Ser1292 phosphorylation levels in G2019S and R1441C knockin brain areas. (**a**) Representative western blots of R1441C knockin (RCKI), wild-type (WT) and G2019S knockin (GSKI) lysates from midbrain, striatum, and cortex of 1, 6, and 12-month-old animals (**b**-**d**). Quantifications of Ser935 phosphorylation (phospho/total LRRK2) and total Lrrk2 levels (Lrrk2/β-actin) in midbrain (**b**), striatum (**c**), and cortex (**d**) at 1, 6, and 12-month-old mice. Each dot in the bar graph represents one animal. Data are shown as mean ± SEM; One-way ANOVA with Dunnett post-hoc test (ns: not significant; * *p* < 0.05; ** *p* < 0.01; *** *p* < 0.001).

Phosphorylation levels of Ser935 and Ser1292 are a readout of LRRK2 activity, and pSer935 is widely used to assess on-target LRRK2 inhibitor engagement [17]. While pSer935 and pSer1292 have been detected in mouse brains [7,18], a systematic comparison across different LRRK2 mutations, brain regions and ages has not been performed. To this aim, we dissected the striatum, cortex, and midbrain of 9 groups of animals (4–5 mice per group) carrying three genotypes (wild-type (WT), KI Lrrk2-G2019S (GSKI), and KI Lrrk2-R1441C (RCKI)) at three different ages (1, 6, and 12 months). To highlight possible differences across genotypes, age-matched WT, GSKI, and RCKI lysates were loaded on the same gel. After western blotting analysis with phospho-Ser1292, phospho-Ser935, and total LRRK2 antibodies (Figure 2a) and subsequent quantification of western blot signals (Figure 2b–d), we made several interesting observations.

First, the levels of phospho-Ser935 in RCKI brains are ~5 times lower than in the WT in all brain areas, in 1 and 6 month-old animals (Figure 2b–d). Instead, at 12 months, the effect is blunted in the cortex, halved in the midbrain but still persistent in the striatum (Figure 2b–d). Second, the levels of phospho-Ser935 in GSKI striatum, midbrain, and cortex are ~50% lower than in WT, in 1 month-old animals and in striatum and midbrain of 6 months-old mice (Figure 2b–d). This result was unexpected given that LRRK2 G2019S from cells display similar Ser935 phosphorylation than LRRK2 WT [9]. At 12 months, Ser935 phosphorylation in the striatum is dramatically reduced to ~10% of the WT, reduced in the cortex but stable in the midbrain. Third, we detected autophosphorylation of Ser1292 only in GSKI, but, quite surprisingly, not in RCKI brains, which behaved as the WT (Figure 2a). This result is in apparent contrast with what has been reported in different cell models where LRRK2 R1441C display increased phospho-Ser1292 (Figure 2a) [3,7]. Of note, in 1-month-old animals phospho-S1292 is detectable only in the striatum, where Lrrk2 expression is higher (Figure 2a).

Last, we observed some differences in Lrrk2 steady state levels across genotypes, but without a consistent trend in one specific region or at a specific age, with the exception of 6 months mice were total Lrrk2 levels are lower in GSKI cortex and midbrain (Figure 2a–d).

### 3.3. Changes in Ser935-Lrrk2, Ser1292-Lrrk2, and Thr73-Rab10 Phosphorylation in G2019S and R1441C Knockin Lungs and Kidneys

**Figure 3:**
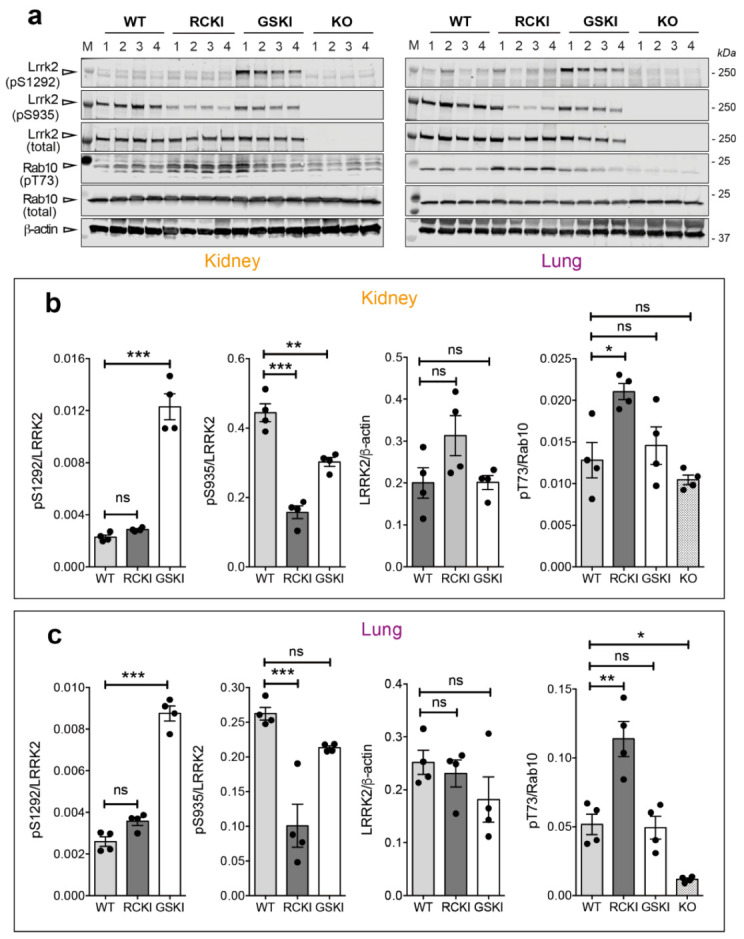
Differences in Ser935-Lrrk2, Ser1292-Lrrk2 and Thr73-Rab10 phosphorylations and total Lrrk2 between G2019S and R1441C knockin lungs and kidneys. (**a**) Representative western blots of WT, RCKI, GSKI, and knockout (KO) lysates from kidney and lung of 12-months-old animals showing Lrrk2 and Rab10 phosphorylation levels. (**b**,**c**) Quantifications of Ser935, Ser1292 phosphorylations (phospho/total Lrrk2), total Lrrk2 (Lrrk2/β-actin), and Thr73-Rab10 phosphorylation (phospho/total Rab10) in kidneys and lungs respectively of WT, RCKI, GSKI mice. Each dot in the bar graph represents one animal. Data are shown as mean ± SEM; One-way ANOVA with Dunnett post-hoc test (ns: not significant; * *p* < 0.05; ** *p* < 0.01; *** *p* < 0.001).

Based on the unexpected finding that R1441C KI brains display undetectable Ser1292 autophosphorylation, we next asked whether this phenotype is restricted to the brain or whether it also applies to other tissues. Because lungs and kidneys have been previously shown to express high levels of LRRK2 [19,20,21] and LRRK2 inhibitors cause morphological changes in type II pneumocytes [22], we evaluated LRRK2 phosphorylation in these tissues isolated from 12 months-old WT, RCKI, GSKI, and Lrrk2 KO mice. After western blot analysis, we observed that pSer1292 is ~4 times higher in GSKI compared to WT, while RCKI lungs and kidneys exhibit similar phospho-Ser1292 levels as the WT (Figure 3a–c), confirming the previous results in brains (Figure 2).

To understand whether the lack of phospho-Ser1292 activation in RCKI mice reflects a general inability of this mutation to manifest a similar gain of kinase function observed in cell models [7], we evaluated Rab10 phosphorylation at Thr73 [3,9,23,24]. Strikingly, Rab10 phosphorylation is significantly higher in RCKI lungs and kidneys compared to WT (Figure 3a,b). Instead, lung and kidney from GSKI mice display levels of Rab10 phosphorylation similar to WT, overall suggesting that autophosphorylation and Rab10 phosphorylation are uncoupled events and may constitute independent readouts of LRRK2 activation. Of note, we could not convincingly detect Rab10 phosphorylation in any brain regions under endogenous (WT and KI) Lrrk2 expression. Finally, the degree of Ser935 phosphorylation in GSKI kidneys, and to a lesser extent in lungs, is in between the level of WT and RCKI conditions, confirming the trend observed in brain (Figure 3a–c).

### 3.4. Age-Dependent Increase of Lrrk2 Phosphorylation and Steady State Levels in BAC-Lrrk2 G2019S Brains

**Figure 4:**
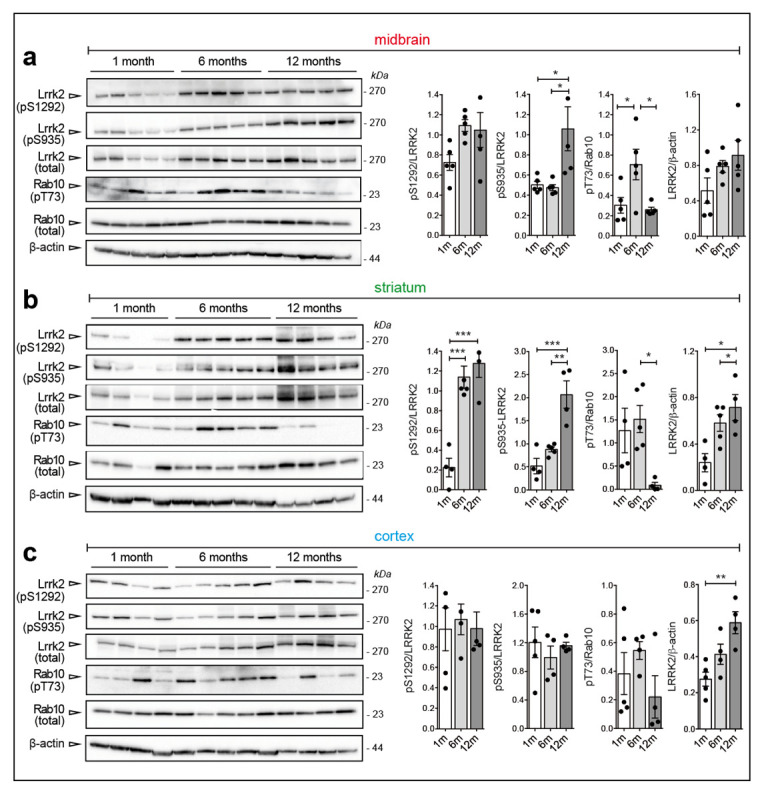
Age-dependent increase of Lrrk2 phosphorylation and steady state levels in BAC-Lrrk2 G2019S brains. (**a**) Representative western blots and relative quantifications of Ser935, Ser1292 phosphorylations (phospho/total Lrrk2), total Lrrk2 (total Lrrk2/β-actin), and Thr73-Rab10 phosphorylation (phospho/total Rab10) in midbrain, (**b**) striatum, and (**c**) cortex of murine BAC-G2019S mice at different ages (1, 6, and 12-months). Each dot in the bar graph represents one animal. Data are shown as mean ± SEM; One-way ANOVA with Dunnett post-hoc test (* *p* < 0.05; ** *p* < 0.01; *** *p* < 0.001).

We next examined the effect of the G2019S mutation in a different mouse model, namely the hemizygous BAC murine Lrrk2 G2019S (mBAC G2019S) mouse. This model, which was developed by Zhenyu Yue and collaborators (Mount Sinai, US), displays ~six-fold higher Lrrk2 expression compared to the endogenous locus [25] and it was shown to develop an age-related decline in striatal dopamine content [25] and 20% loss of TH neurons at 20 months of age [26]. Due to the large differences in Lrrk2 expression between WT and mBAC G2019S mice, we did not compare the genotypes but rather examined Lrrk2 and Rab10 phosphorylation in mBAC G2019S comparing striatum, cortex and midbrain at different ages (5 mice per age). Of note, while phospho-Thr73 Rab10 could not be robustly detected in brains with endogenous Lrrk2 expression (data not shown), it is clearly detectable in mBAC G2019S brains (Figure 4a–c).

**Figure 5:**
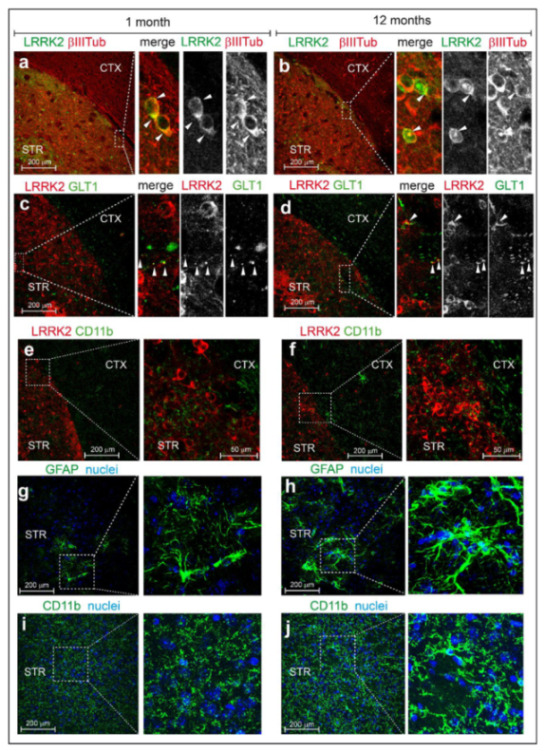
Immunofluorescent analysis of Lrrk2 distribution and subcellular localization in mBAC-Lrrk2-G2019S brain. Representative co-immunofluorescent staining of brain slices from hemizygous transgenic mice overexpressing murine BAC-Lrrk2-G2019S. In detail: (**a**,**b**) tot-Lrrk2 (green) distribution in cortex and striatum and subcellular localization in β-III-tubulin positive neurons (red) of 1-month-old and 12-months-old mice; (**c**,**d**) tot-Lrrk2 (red) distribution in cortex and striatum and subcellular localization in GLT-1 positive astrocytes (green) of 1-month-old and 12-months-old mice; (**e**,**f**) tot-Lrrk2 (red) distribution in cortex and striatum and subcellular localization in CD11b positive microglia (green) of 1-month-old and 12-months-old mice. Representative immunofluorescent staining of (**g**,**h**) GFAP and (**i**,**j**) CD11b-positive cells highlighting the different shape and activation level of striatal astrocytes and microglia respectively, at 1 and 12 months of age. *n* = 3 animals each staining. Scale bars 200 μm, and 50 μm.

In this dataset, we observed that: (i) LRRK2 expression increases upon aging in striatum and cortex; (ii) pSer935 and pSer1292 increase with aging in the striatum; (iii) Rab10 phosphorylation decreases at 12 months when Lrrk2 expression peaks, further supporting the notion that autophosphorylation and Rab10 phosphorylation are uncoupled events, at least in this context (Figure 4a–c). The observed differences in endogenous Lrrk2 levels across brain regions (Figure 1) are not as pronounced in the BAC G2019S mouse model (Figure S1), suggesting that Lrrk2 overexpression may blunt these tissue-specific variations. Finally, we prepared coronal brain slices from 1 and 12-month-old mBAC G2019S mice to evaluate Lrrk2 expression and phosphorylation at the cellular level with MJFF2(c41-2) and UDD210(12) antibodies, respectively. As shown in Figure 5a–f and Figure S3, total and phospho-Lrrk2 antibodies give high and specific signals (Figure S2) in the striatum, while the labeling in the cortex is much weaker (Figure S4).

We also observed that Lrrk2 (MJFF2) partially colocalizes with *β*-III-tubulin, a neuronal marker (Figure 5a,b) and to a lesser extent with GLT-1, a major glutamate transporter mainly expressed by astrocytes (Figure 5c–d). Instead, no co-localization with the microglial marker CD11b was observed (Figure 5e,f). Quite interestingly, we further noticed that phospho-Lrrk2 (phospho-Ser935) colocalizes with *β*-III-tubulin but no (or very faint) co-localization with GLT1 could be observed (Figure S3), suggesting that LRRK2 Ser935 phosphorylation is higher in neurons than in astrocytes. Given the age-dependent increase in expression of Lrrk2 in BAC-G2019S mice (Figure 4), we wondered whether this is paralleled to an increase in gliosis, a pathological feature reported in mutant LRRK2-associated PD [27]. To this end, coronal slices from 1 and 12-months-old BAC-G2019S mice were stained for GFAP, an intermediate cytoskeletal protein whose expression increases during astrogliosis [28]. As shown in Figure 5g,h, striatal astrocytes from 12-months-old mice exhibit a more ramified morphology as compared to 1-month-old animals, overall pointing to enhanced gliosis in aging BAC-G2019S brains. In addition, we observed a more intense CD11b signal as well as the presence of ramified microglial cells in cortico-striatal slices from 12 month-old BAC-G2019S mice, overall pointing at an increased inflammatory state in aged BAC-G2019S brains (Figure 5i,j).

## 4. Discussion

**Figure 6:**
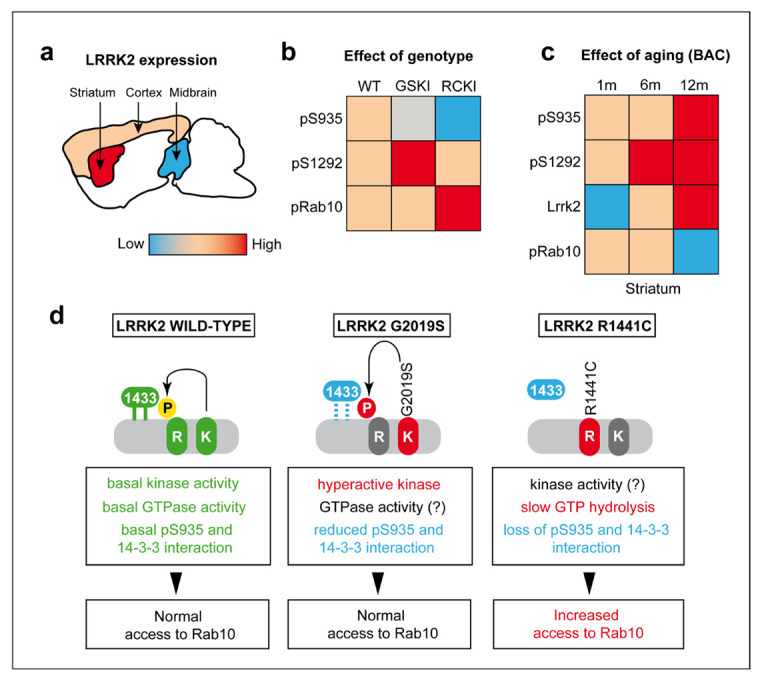
The differential impact of LRRK2 mutations on Rab10 phosphorylation in brain. (**a**) Schematic representation of LRRK2 protein levels in cortex, midbrain, and striatum. (**b**) LRRK2 and Rab10 phosphorylation levels as function of LRRK2 common mutations (G2019S and R1441C) and (**c**) as function of ageing in murine BAC-Lrrk2-G2019S mice. (**d**) Schematic summary of the possible molecular mechanisms underlying the different levels of Rab10 phosphorylation in presence of wild-type or mutated LRRK2. In detail, our data suggest that in the presence of the R1441C-LRRK2 mutation, the reduced phosphorylation at Ser935 may lead to a reduction of LRRK2-14-3-3s binding and thus to an increased access to Rab10.

Understanding the effects of pathogenic mutations on LRRK2 phosphorylation and cellular activity is critical to clarify their impact on LRRK2 signaling and to identify mutation-specific effects important for disease diagnosis. There is general consensus that both kinase and non-kinase mutations increase LRRK2 activity towards its major substrates, namely LRRK2 itself and a subset of RAB GTPases [3,7]. However, most of these studies use cell models with protein overexpression or primary cells isolated from KI mice [3,7]. In contrast, the impact of mutations in a complex environment such as the brain where different cell types constantly exchange information, is poorly known. Here, by comparing LRRK2 mouse-models brains and peripheral tissues carrying two major mutations, namely G2019S in the kinase domain and R1441C in the ROC/GTPase domain, we found that heterologous phosphorylation (Ser935), autophosphorylation (Ser1292), and substrate phosphorylation (Rab10) have patterns not strictly predicted by cellular studies. First, we observed that Lrrk2 is highly expressed in the mouse striatum, while the protein shows significantly lower levels in the midbrain (Figure 6a).

High levels of LRRK2 in the striatum were previously reported [15,16], as well as specific functions in striatal medium spiny neurons [29,30]. Of interest, in all the models analyzed we found the clearest effects in the striatum. GSKI mice exhibit an age-dependent decline in Ser935 phospho-levels (Figure 6b), while increased levels and phosphorylation of Lrrk2 with aging are found in BAC-G2019S mice overexpressing the endogenous murine Lrrk2 locus (Figure 6c). In BAC-G2019S mice, the kinase is expressed in neurons and in some GLT1-positive structures, consistent with the previously reported expression in astroglia [31,32]. Not all LRRK2 positive cell bodies co-localize with the neuronal specific marker β-III-tubulin, supporting LRRK2 expression also in non-neuronal cells [31,32].

By comparing LRRK2 and Rab10 phosphorylation in KI G2019S and R1441C brains and lung/kidney, we made two major observations. First, the G2019S mutation increases autophosphorylation at Ser1292 while the R1441C mutation does not (Figure 6b). Second, R1441C but not G2019S results in increased phospho-Rab10, at least in kidneys and lungs where detection of phospho-Rab10 was possible (Figure 6b). The lack of phospho-Rab10 detection in the brain may be explained by the high expression levels in this tissue of PPM1H, a phosphatase selectively dephosphorylating Rab GTPases [11]. Furthermore, the R1441C mutation dramatically reduces Ser935 phosphorylation (Figure 6b), in line with cellular studies [5,6], instead the phosphorylation tone of Ser935 in GSKI is in between WT and RCKI (Figure 6b). The R1441 residue is located in the GTPase/ROC domain and substitution of the arginine with a cysteine decreases GTP hydrolysis [33] by locking the GTPase in its GTP-bound monomeric conformation, thereby trapping LRRK2 in an “on” conformation [34,35]. From our analysis, Ser935 phosphorylation is dramatically reduced in brain, lung, and kidney of RCKI mice, consistent with cellular studies [5,6]. Phosphorylation of Ser935 and Ser910 is necessary for the binding of 14-3-3 proteins, which results in LRRK2 diffuse cytoplasmic localization, at least under overexpression conditions [5]. In contrast, dephosphorylation of these residues by the action of LRRK2 phosphatases or via PAK6 phosphorylation of 14-3-3s, causes LRRK2 to compartmentalize [36,37]. In agreement, the basal dephosphorylated state of LRRK2 R1441C in Ser935 is coupled to a compartmentalized phenotype in cells, possibly indicating that the kinase is constitutively localized in its signaling domains [6,38]. Recent studies showed that membrane-bound RAB29 recruits and activates LRRK2 via its ankyrin (ANK) region which is near the cluster of serins that contains Ser935 [39,40,41]. Purlyte et al. further observed that pathogenic LRRK2 mutants with increased GTP binding (e.g., R1441G and Y1699C) display higher levels of Ser1292 autophosphorylation than wild-type LRRK2, but that the already higher levels of basal phospho-RAB10 are not further increased upon RAB29 expression [39], overall suggesting that ROC mutants may be constitutively recruited at RAB10-positive membranes domains independently on RAB29. This model would also explain why low Ser935 phosphorylation in LRRK2 R1441C still guarantees RAB10 phosphorylation, given that phospho-Ser935 is normally required for RAB29 recruitment of LRRK2 [39]. Further supporting these conclusions, a recent study also from Alessi’s group [42] reported that Rab29 KO in R1441C KI mice does not reduce the elevated Rab10 phosphorylation in MEFs or tissues, again suggesting that endogenous Rab29 is not sufficient to explain the elevated activity of the R1441C pathogenic mutant. This model fits with another observation by the same authors showing that RAB29-deficient binding mutants in the ANK domain are devoid of Ser935 phosphorylation but can still promote LRRK2-mediated RAB10 phosphorylation [39]. It remains to be explained why the R1441C mutation results in increased Ser1292 autophosphorylation in cells overexpressing this mutant form of LRRK2 but not in brain and peripheral tissues isolated from mice (Figure 6b). One obvious difference is that we are comparing overexpression versus endogenous expression, which should warn on interpreting overexpression studies with caution. Another possibility is that humans and mice possess different regulatory mechanisms or expression levels of LRRK2 and its interactors/substrates, which would result in different cellular outcomes.

Taking these and our findings together, we can postulate that GTP-locked LRRK2 R1441C does not need to undergo autophosphorylation in vivo being “constitutively” localized at RAB10-membranes (or other compartments), bypassing RAB29-mediated recruitment and activation (Figure 6d). The consequence of a chronic RAB10 hyperphosphorylation could result in reduced ciliogenesis, thus impacting on brain functionality as suggested by recent works [43,44]. Future studies addressing the precise consequences of R1441C-dependent RAB10 hyperphosphorylation on ciliogenesis, as well as on lysosomal function [41,45], will better clarify the pathogenic mechanisms of this mutation.

Our study further highlights that LRRK2 mutations located in different domains (i.e., GTPase and kinase) likely operate through different pathogenic mechanisms. Knock-in mice carrying the G2019S mutation at the endogenous locus exhibit increased autophosphorylation at Ser1292 but Rab10 phosphorylation levels are similar to wild-type (Figure 2 and Figure 6b–d). In contrast with the R1441C mutation which does not affect *per se* the catalytic properties of the kinase, the G2019S mutation located in the activation loop of the kinase increases activity by doubling the Vmax [46]. Based on the above considerations, we can predict that the higher autophosphorylation observed is a direct consequence of a kinase intrinsically more active, an outcome that is also observed in cellular models [3,7,47]. The fact that this hyperactive kinase does not hyper-phosphorylate Rab10 can be explained by the fact that LRRK2 G2019S requires RAB29 recruitment to RAB10-containing membranes similarly to wild-type LRRK2, as shown by Purtyle and collaborators [39] (Figure 6d). This implies that hyper-phosphorylation of other autophosphorylation sites located in the ROC domain [4] does not result in a GTP-locked protein but rather provide some fine regulation whose effect is not captured when assessing phospho-Rab10.

Translating these findings into therapeutic implications, kinase inhibitors are predicted to normalize the intrinsically higher kinase activity of LRRK2 G2019S carriers. While R1441C carriers may also benefit from a kinase inhibitor-based therapy through “on site” reduction of substrate hyperphosphorylation (i.e., Rab10), strategies that can correct the lower GTP hydrolysis of RocCOR mutants may represent an alternative, and possibly more effective, therapeutic approach for LRRK2-R1441C carriers. Another implication of the different mode of action for these two mutations is that they may require distinct pharmacodynamic readouts to assess on-target kinase inhibition: dephosphorylation of Ser935/Ser1292 for G2019S carriers and Rab10 dephosphorylation for R1441C carriers.

One intriguing observation from our analysis is that phosphorylation of Ser935 in GSKI brain lung and kidney is reduced by ~half. These results were not observed in cell lines [9] but are in line with two recent papers where pSer935 was reported to be reduced in peripheral blood mononuclear cells (PBMCs) of disease-manifesting G2019S carriers compared to idiopathic PD or controls [48,49], overall supporting the GSKI mouse with endogenous mutant Lrrk2 expression as a valuable pre-symptomatic model of disease. Since overexpression of RAB29 was observed to reduce Ser935 phosphorylation in both WT and G2019S LRRK2 [39], one possibility is that the decreased phospho-Ser935 observed in GSKI tissues is due to increased RAB29-dependent recruitment. If this is true, the lack of increase in phospho-Rab10 may suggest that LRRK2 G2019S is recruited to subcellular membrane compartments that are Rab10 negative (Figure 6d). Further studies comparing in vivo the effect of G2019S and R1441C mutations against different RAB GTPases may reveal how different mutations impact different cellular processes.

In line with a previous study by Mercatelli and collaborators [50], we also observed reduced total Lrrk2 levels in cortex and midbrain of GSKI compared to WT in 6 month-old animals (Figure 2). The reasons for this decline are unclear, although it could be speculated that it represents a neuronal response to downregulate the excessive kinase activity associated with mutant Lrrk2, a compensatory mechanism that is lost in aged mice (12 months), consistent with the late onset of PD pathology. Instead, the R1441C mice show a mild decline in total Lrrk2 levels at 12 months of age (Figure 2), further evidencing how the two mutations manifest with distinct phenotypes.

Finally, by comparing the effect of aging on Lrrk2 and Rab10 phosphorylation in the context of BAC-Lrrk2-G2019S overexpressing mice, we collected further evidence that Ser1292 autophosphorylation and Rab10 phosphorylation are uncoupled phenomena. Specifically, while Lrrk2 levels and phosphorylation increase with aging (up to 12 months), phosphorylation of Rab10 decreases at 12 months, with the strongest effect observed in the striatum (Figure 6c). The reasons behind this effect are unclear. One possibility could be that the levels of PPM1H increase with aging to counteract the parallel increase in Lrrk2 observed. Intriguingly, a recent study found a genetic association between PPM1H and INFα levels in systemic lupus erythematosus (SLE) patients [51]. As also LRRK2 can modulate the risk of inflammatory diseases such as SLE [52,53] and neuroinflammation is increased in 12-month old BAC G2019S mice (Figure 5), future studies should be directed at exploring the possible link between LRRK2 and PPM1H in PD-related inflammation. While additional investigations are clearly required, these findings together with the previous observations in KI mice support the importance of encoding the effects of different LRRK2 mutations in specific tissues and at specific ages.

PD is a multisystemic disorder for which no cure is available. LRRK2 inhibitors are under clinical development and phase I clinical trials have already proved efficacy and safety [54]. However, LRRK2-PD neuropathology is variable, ranging from typical Lewy bodies pathology, to pure nigral degeneration, Tau pathology, and progressive supranuclear palsy-like pathology [27], which may reflect the different mode of action of different mutations in combination with other genetic and environmental factors. Thus, inhibition of LRRK2 activity with kinase inhibitors may work well with G2019S patients but may be less effective against mutations impairing GTP hydrolysis. A clear understanding of the impact of LRRK2 pathogenic mutations on autologous and heterologous LRRK2 phosphorylation in peripheral tissues from patients not only appears valuable as a predictive biomarker of disease but could also provide the mechanistic knowledge to develop personalized treatments for different LRRK2-PD.

## Supplementary Materials

The following are available online at https://www.mdpi.com/2073-4409/9/11/2344/s1, Figure S1: Total LRRK2 steady-state levels in mBAC-G2019S PD-relevant brain areas, Figure S2: Immunofluorescent staining of brain slices from hemizygous transgenic mice overexpressing murine BAC-Lrrk2-G2019S and Lrrk2-KO mice, Figure S3: Immunofluorescent staining of brain slices from hemizygous transgenic mice overexpressing murine BAC-Lrrk2-G2019S mice, Figure S4. Immunofluorescent staining of brain slices from hemizygous transgenic mice overexpressing murine BAC-Lrrk2-G2019S mice.
