# Supplementary material for: Divergent Effects of G2019S and R1441C LRRK2 Mutations on LRRK2 and Rab10 Phosphorylations in Mouse Tissues
Source: Cells. 2020 Oct 22;9(11):2344. doi: 10.3390/cells9112344 (PMC7690595; doi:10.3390/cells9112344)
Supplement: Supplementary file 1 [file cells-09-02344-s001.pdf]

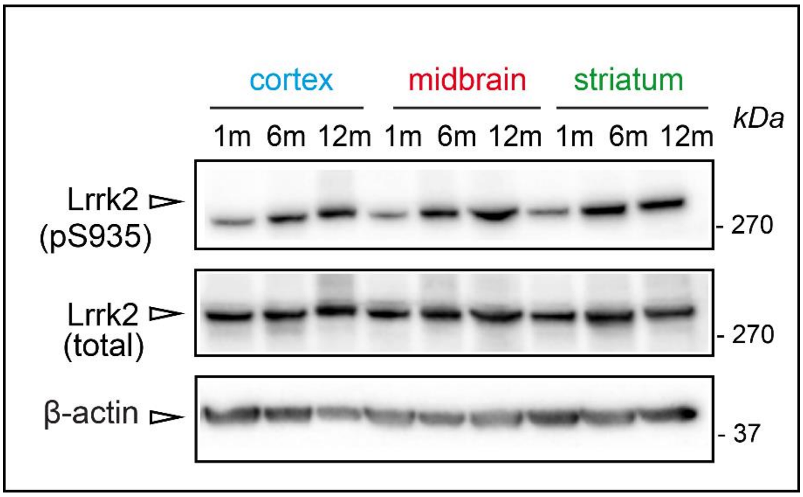

Figure S1. Total LRRK2 steady-state levels in mBAC-G2019S PD-relevant brain areas. Representative Western Blot showing Lrrk2 steady state levels in midbrain, striatum and cortex at 1, 6 and 12-month-old mice.

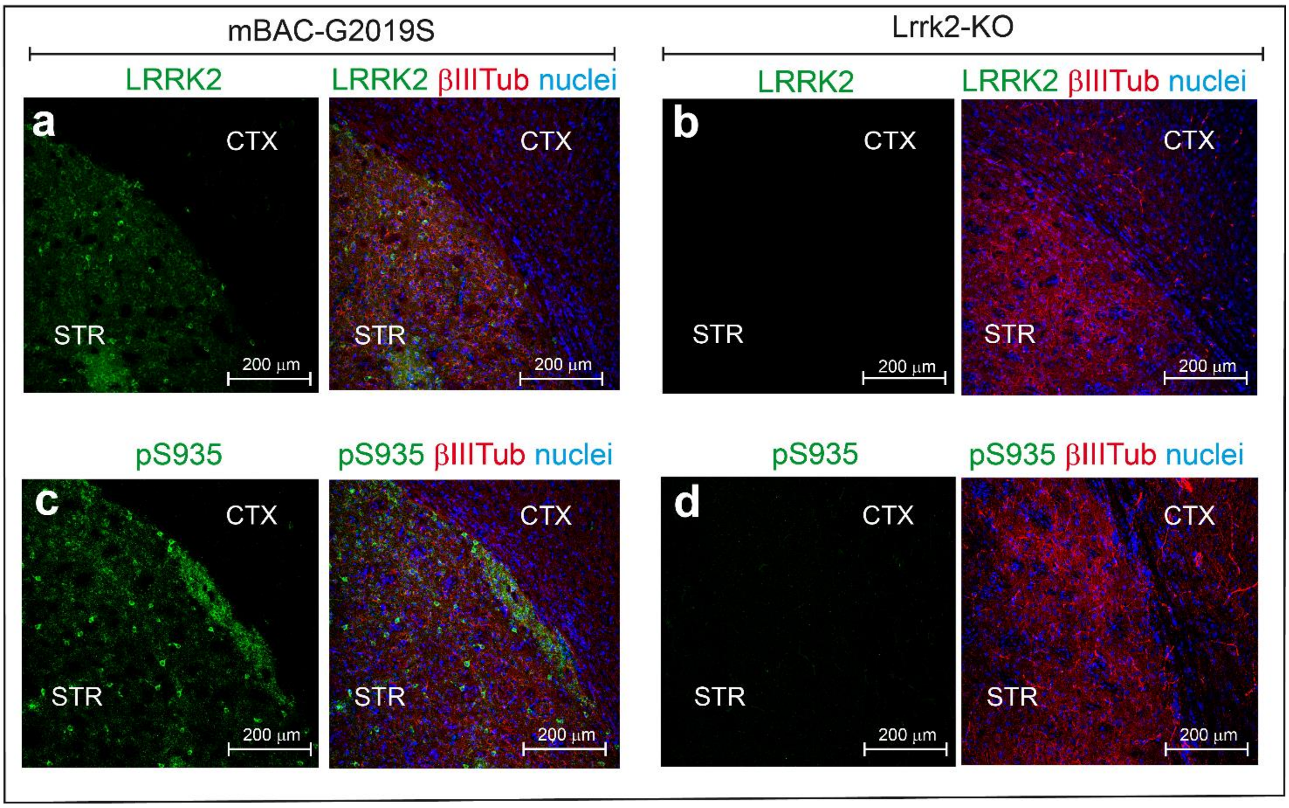

Figure S2. Immunofluorescent staining of brain slices from hemizygous transgenic mice overexpressing murine BAC-Lrrk2-G2019S and Lrrk2-KO mice. (**a,b**) Representative staining of brain slices from BAC-Lrrk2-G2019S mice or (**b,d**) Lrrk2-KO mice with MJFF2(c41-2) and UDD210(12) antibodies showing the specificity of the staining.

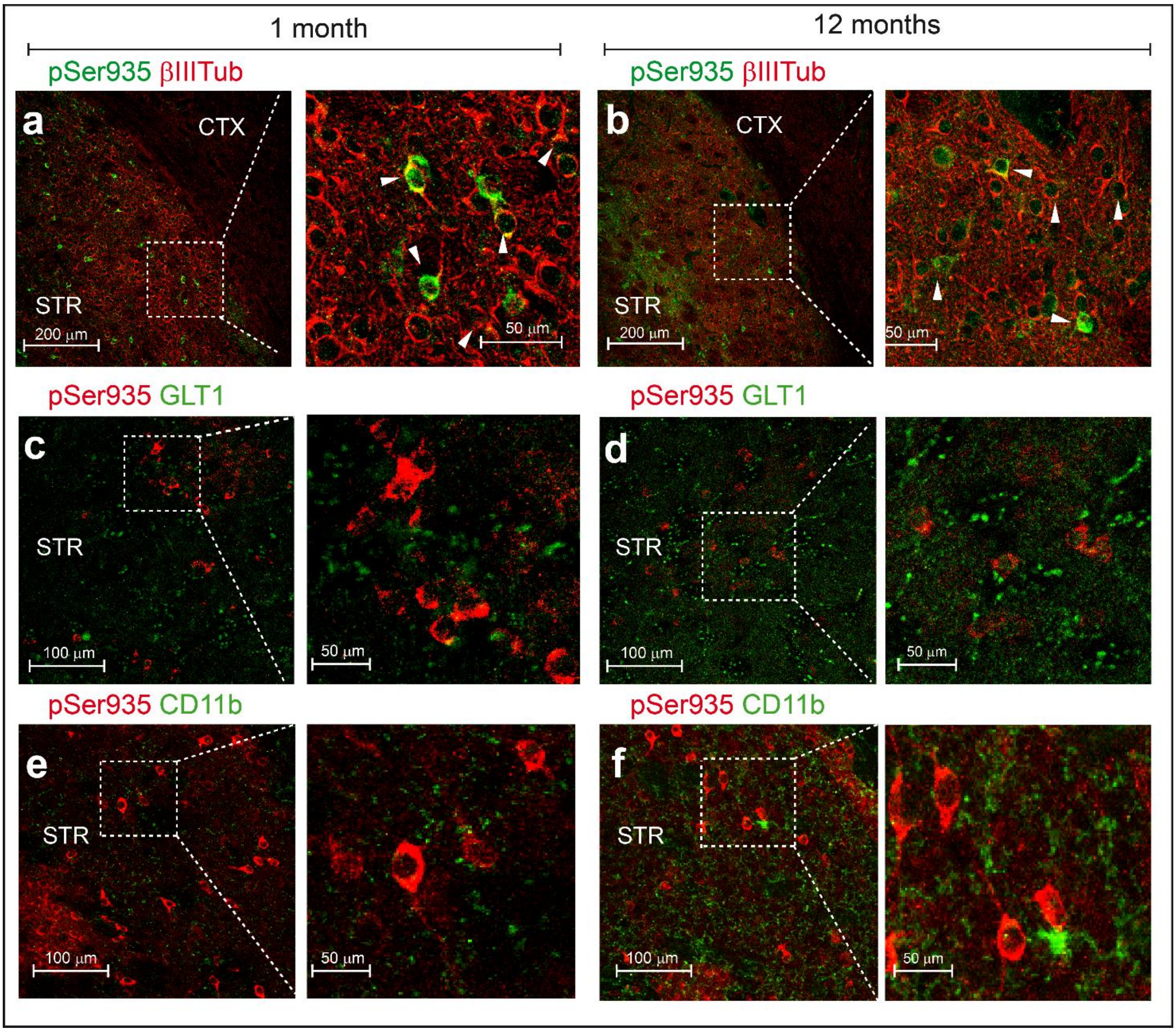

Figure S3. Immunofluorescent staining of brain slices from hemizygous transgenic mice overexpressing murine BAC-Lrrk2-G2019S mice. (**a**–**f**) Representative staining of brain slices from BAC-Lrrk2-G2019S mice with anti-phosphoSer935 of LRRK2 antibody showing co-localization only in b-III-tubulin-positive cells.

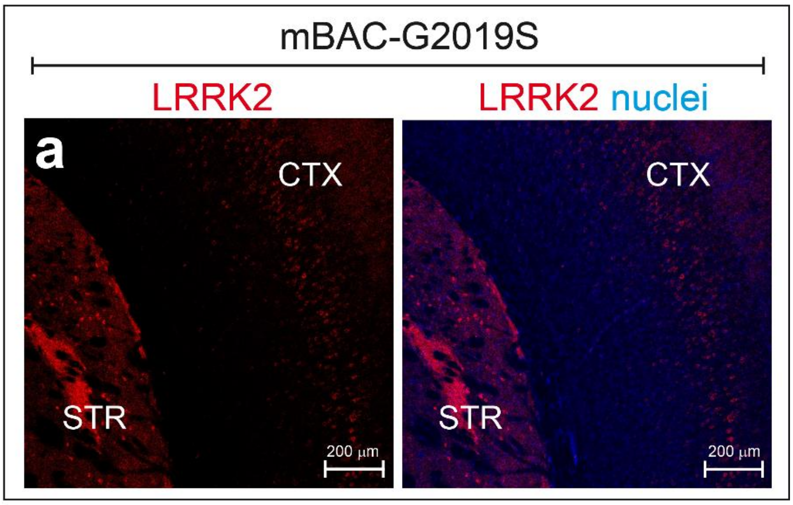

Figure S4. Immunofluorescent staining of brain slices from hemizygous transgenic mice overexpressing murine BAC-Lrrk2-G2019S mice. Representative staining of brain slice from BAC-

Lrrk2-G2019S mice with anti-LRRK2 MJFF2(c41-2) antibody showing the differential expression of Lrrk2 in the cortex.
